# Evaluation of optimized bronchoalveolar lavage sampling designs for characterization of pulmonary drug distribution

**DOI:** 10.1007/s10928-015-9438-9

**Published:** 2015-08-28

**Authors:** Oskar Clewe, Mats O. Karlsson, Ulrika S. H. Simonsson

**Affiliations:** Department of Pharmaceutical Biosciences, Uppsala University, Uppsala, Sweden

**Keywords:** Bronchoalveolar lavage, Pulmonary distribution, Sampling design, Pharmacometrics

## Abstract

Bronchoalveolar lavage (BAL) is a pulmonary sampling technique for characterization of drug concentrations in epithelial lining fluid and alveolar cells. Two hypothetical drugs with different pulmonary distribution rates (fast and slow) were considered. An optimized BAL sampling design was generated assuming no previous information regarding the pulmonary distribution (rate and extent) and with a maximum of two samples per subject. Simulations were performed to evaluate the impact of the number of samples per subject (1 or 2) and the sample size on the relative bias and relative root mean square error of the parameter estimates (rate and extent of pulmonary distribution). The optimized BAL sampling design depends on a characterized plasma concentration time profile, a population plasma pharmacokinetic model, the limit of quantification (LOQ) of the BAL method and involves only two BAL sample time points, one early and one late. The early sample should be taken as early as possible, where concentrations in the BAL fluid ≥ LOQ. The second sample should be taken at a time point in the declining part of the plasma curve, where the plasma concentration is equivalent to the plasma concentration in the early sample. Using a previously described general pulmonary distribution model linked to a plasma population pharmacokinetic model, simulated data using the final BAL sampling design enabled characterization of both the rate and extent of pulmonary distribution. The optimized BAL sampling design enables characterization of both the rate and extent of the pulmonary distribution for both fast and slowly equilibrating drugs.

**Electronic supplementary material:**

The online version of this article (doi:10.1007/s10928-015-9438-9) contains supplementary material, which is available to authorized users.

## Introduction

To combat and prevent further rise in antibiotic resistance, antibiotic dosing regimens needs to be based on pharmacokinetics (PK) and pharmacodynamics (PD). Direct measurements of antibiotic concentrations close or at the site of infection as opposed to plasma concentrations have been promoted for antibiotics due to possible differences in distribution to various tissues. The distribution to the site of action from plasma will directly have impact on the relationship between concentrations in plasma and concentrations at the site of action. Basic pharmacodynamic principles further dictate that the observed drug effect is directly dependent on the drug concentration. Thus, if the drug carries out its effect in a tissue other than where drug concentration is measured, the possibility of a discrepancy between measured concentration and observed effect exists. The effect could then potentially be better correlated to the concentration at the site of action. This possibility is one of the reasons behind the development of methods allowing for quantification of drug concentrations close to or at the site of action, in order to possibly better be able to describe exposure–response relationships.

Bronchoalveolar lavage (BAL) is a semi-invasive method used in both research and clinical practice as a way of quantifying drug concentrations from epithelial lining fluid (ELF) and alveolar cells (AC) [1–5]. For pulmonary infections, concentrations of antibiotics in ELF for extracellular pathogens and alveolar macrophage (AM) cells for intracellular pathogens have for example been proposed to reflect antibiotic activity in pneumonia [4]. Capturing the drug concentration ratio between plasma and ELF or AC is thus of importance in order to guarantee that sufficient drug concentrations reach the pulmonary tract. It is however important not only to characterize the extent of distribution to ELF or AC but also characterize the rate of distribution from plasma when obtaining relationships between PK and PD. This is especially relevant for drugs and compounds without an instantaneous or fast equilibrium between plasma and the lung, where the exposure in plasma may not be a good marker of the drug exposure at the site of action. This could potentially lead to a distorted PKPD relationship.

In a review by Rodvold et al [3], the penetration of various anti-infective agents into ELF was summarized. One of the conclusions in the review was that many studies involving BAL sampling are not designed to enable description of both the extent and rate of distribution of drug concentrations from plasma to pulmonary tissue. Due to the semi-invasive nature of the BAL method, only one or two samples is often taken from each subject. This results in that it is impossible to describe a full distribution profile from each subject. One way of dealing with this is by dividing the study population into subgroups and conduct sampling of these subgroups at different times after dose [6–8]. This approach compared to the single sample approach, that only provides a snapshot of the distribution at the time of sampling, enables a potential characterization of both the rate and extent of distribution. Both methods further try to capture both the peak concentration and the minimum concentration in ELF or AC. This to maximize the information gained when using the quantified concentrations in plasma, ELF or AC to calculate concentration ratios or exposure. Both sampling methods however require previous knowledge regarding the pulmonary distribution of the drug to capture both the peak and the minimum drug concentrations. For a novel compound, where nothing or very little is previously known with regards to its pulmonary distribution, these sampling strategies will be difficult to implement due to that the time of the peak and minimum ELF and AC concentrations are unknown. Important to realize is also that a study design capturing only point estimates of concentrations cannot be used for simulation purposes. In a publication by Clewe et al [9], a pharmacometric model enabling characterization of both the rate and extent of drug distribution from plasma to ELF and AC was developed using rifampicin (RIF) as an example. The model was developed on single time point estimate data and in the publication limitations, with regards to this kind of data, in describing the rate of distribution from plasma is discussed. The data used in the publication by Clewe et al [9], consisting of RIF concentrations quantified in ELF and AC at approximately 4 h and in plasma at 2 and 4 h after dose, contained no information to enable a correct characterization of the distribution rates from plasma to ELF and AC. Thus forcing the assumption of instantaneous distribution. A similar model structure as the general pulmonary distribution model has been presented earlier in an example of drug distribution to pulmonary lesions in rabbit [10]. The general pulmonary distribution model applied in this work [9] constitutes an approach for characterizing the ratio (extent) and rate of distribution to ELF and/or AC which is not dependent on an individual rich pharmacokinetic BAL sampling or sampling at many different time points between subjects. The approach is further to be viewed as drug unspecific as the general pulmonary distribution model can be linked to any type of plasma PK model, not only to the plasma PK model used as an example in the publication by Clewe et al [9].

Modeling and simulation has previously been successfully used to provide information on aspects related to study design [11, 12] and should in the field of biomedical science now be considered as an integral part of research and development [13, 14]. Evaluation in a clinical data setting cannot be used for validation of the approach. Validation of the approach is commonly done using simulation and estimation techniques [11, 12]. In such an approach the simulations are made for different designs and the parameter estimates that are re-estimated are benchmarked against the true parameter estimates. In a clinical data setting, the true parameter estimates are never known and bias and precision given a specific design cannot be evaluated. The aim of this work was thus to develop and evaluate a general optimized BAL sampling design, making use of a previously published pharmacometric modeling approach for describing pulmonary distribution [9], that would allow for characterization of both the rate and extent of distribution from plasma to ELF or AC for two hypothetical drugs with different distribution rates (fast and slow). The optimization of the sampling design does however not make use of the concept of optimal design theory for non-linear mixed effects models [15, 16], which involves some type of optimality criteria and maximization of the Fisher information matrix (FIM). Relative bias and relative root mean square error (rRMSE) in the parameter estimates were evaluated using simulations for different number of samples per subject (1 or 2) and total sample size.

## Materials and methods

A previously developed pharmacometric modeling approach enabling characterization of pulmonary distribution in the form of rate and ratio (extent) of distribution from plasma to ELF and AC [9] was used as a basis for the sampling design evaluation. The previously published modeling approach was developed using RIF plasma and BAL data and hence consisted of a RIF plasma PK model [17] and RIF specific plasma to ELF and AC distribution models describes as:

1 $$\frac{{dC_{ELF} }}{dt} = k_{ELF} \times \left( {R_{ELF/plasma} \times \frac{{A_{plasma} }}{{V_{plasma} }} - C_{ELF} } \right)$$

2 $$\frac{{dC_{AC} }}{dt} = k_{AC} \times \left( {R_{AC/plasma} \times \frac{{A_{plasma} }}{{V_{plasma} }} - C_{AC} } \right)$$

where *C* is concentration, *k*_*ELF*_ is the distribution rate constant for the transfer of drug from plasma to ELF, *R*_*ELF/plasma*_ is the ELF/plasma concentration distribution ratio (extent), *k*_*AC*_ is the distribution rate constant for the transfer of drug from plasma to AC and *R*_*AC/plasma*_ is the AC/plasma concentration distribution ratio (extent). *A*_*plasma*_*/V*_*plasma*_ is the concentration of drug predicted in the plasma compartment at time t, with *A*_*plasma*_ being the amount of drug in plasma and *V*_*plasma*_ being the apparent plasma volume of distribution.

**Fig. 1:**
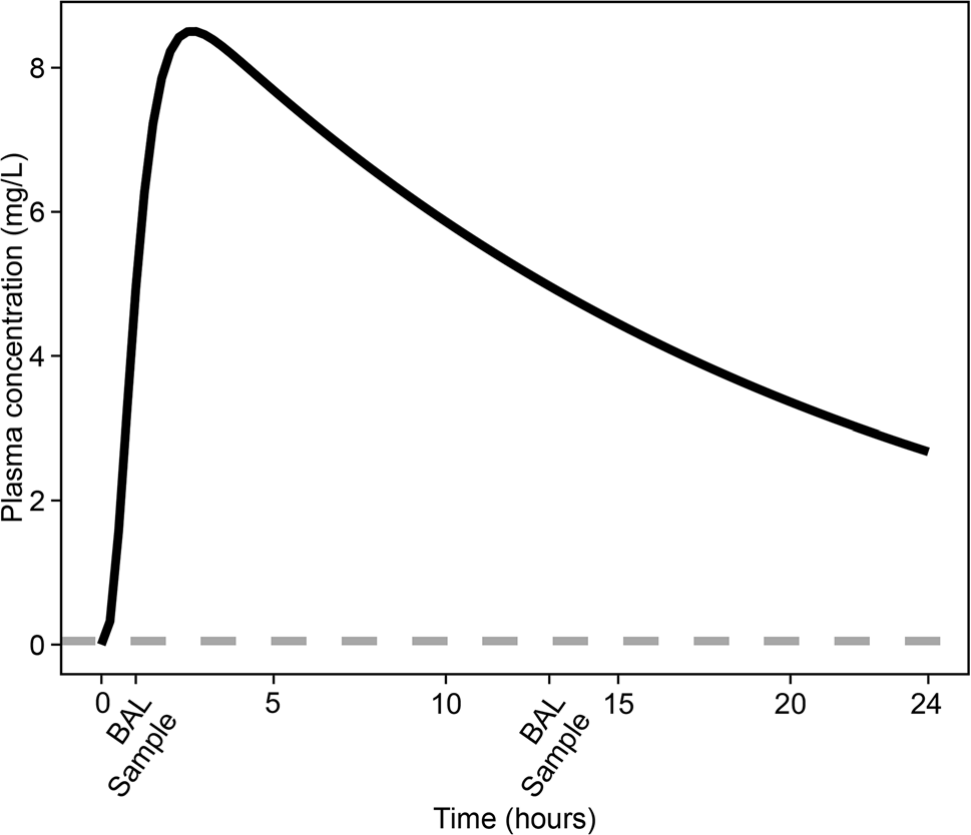
Simulated typical plasma concentrations versus time after a single 600 mg oral dose (*black solid line*) based on final estimates from the population pharmacokinetic model [9]. The *grey dashed line* represents the limit of quantification (LOQ), 0.05 mg/L, of rifampicin in bronchoalveolar lavage (BAL) fluid (epithelial fluid or alveolar cells). The identified optimized rifampicin BAL sampling time points are marked on the x-axis and were 1 and 13 h post dose. The sampling time points should be as early and as late as possible within the study time frame and were selected from the simulated plasma concentration time profile based on correspondence in plasma concentrations; plasma concentrations ≥ LOQ in BAL fluid and maximizing BAL fluid concentrations ≥ LOQ in BAL fluid assuming a slow distribution

**Fig. 2:**
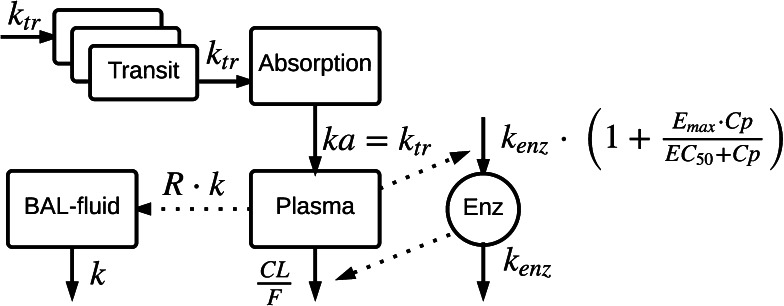
Schematic representation of the pharmacokinetic model [17] and the general pulmonary distribution model [9] used for the simulations and the evaluation of the bronchoalveolar lavage (BAL) sample design. Drug is transferred via a number of transit absorption compartments to the absorption compartment and further via the rate constant *k*_*a*_ to the central plasma compartment. Auto-induction is described with an enzyme turn-over model in which the drug plasma concentration increased the enzyme production rate (*k*_*ENZ*_) which in turn increased the enzyme pool (Enz) in a non-linear fashion by means of an E_MAX_-model. Cp is the drug plasma concentration and *E*_*max*_ is the maximal auto-induction of oral clearance (*CL/F*). *EC*_*50*_ is the drug concentration resulting in 50 % of the maximal auto-induction of *CL/F*. The general pulmonary distribution model includes the distribution rate constant (*k*) for the transfer of drug from plasma to BAL fluid (epithelial lining fluid or alveolar cells). *R* is the BAL fluid/plasma concentration distribution ratio (extent)

The basis for the sampling design was that a maximum of two samples was to be taken from the same individual within a time frame of 24 h. Further, the approach assumed that the studied drug’s plasma concentration profile and the LOQ for the drug in the BAL sample is known. In the publication by Clewe et al [9], a RIF plasma PK model was used an example of a drug plasma PK model. This RIF plasma PK model (Fig. 2) was in this study used as an example of a plasma PK model. Characterization of the typical plasma concentration was done by simulations with the plasma PK model (Fig. 1). The LOQ was set to the values reported (plasma 0.5 and 0.015 mg/L for the BAL sample) for the data [1] used in the publication by Clewe et al [9]. The plasma to ELF and AC distribution was in the model by Clewe et al [9] described separately with two different distribution rate constants and distribution ratios (extents) for ELF and AC (Eqs. 1, 2). In this study only one pulmonary sub-model was used for the evaluation of the optimized sampling design.

3 $$\frac{dC}{dt} = k \times \left( {R \times \frac{{A_{plasma} }}{{V_{plasma} }} - C} \right)$$

In Eq. 3, *C* is concentration in the pulmonary compartment, *k* is the distribution rate constant for the distribution of drug from plasma to the pulmonary compartment, *R* is the pulmonary to plasma concentration distribution ratio (extent). *A*_*plasma*_*/V*_*plasma*_ is the concentration of drug predicted in the plasma compartment at time t, with *A*_*plasma*_ being the amount of drug in plasma and *V*_*plasma*_ being the apparent plasma volume of distribution. This one pulmonary compartment could thus represent either the distribution from plasma to ELF, AC or both. A schematic illustration of the model used for the evaluation of the sampling design is shown in Fig. 2. To illustrate the models ability of handling different distribution scenarios, simulations with different distribution rate constants (k) and different distribution ratios (extents) (R) were performed. The results from the simulations shows that the model well handles different distribution rates (Online Resource 1) and ratios (extents) (Online Resource 2).

Based on the simulated plasma concentration versus time profile (Fig. 1), two time points for the BAL sampling were selected. These two samples needs to be taken at two time points where the plasma concentration is the same i.e. one sample in the raising part of the plasma concentration time profile and one in the declining part of the plasma concentration time profile. In addition, the time points needs to be selected in order to maximize the likelihood of that the BAL concentrations are above the BAL LOQ. Most often, the rate of pulmonary distribution is not known. If the distribution is perfusion limited, the BAL concentration profile will follow the plasma profile in the raising part of the concentration profile. In the case of a distribution rate limitation, the BAL concentration profile may increase slower than the plasma profile and as such, it may take longer time until the BAL concentrations are above the BAL LOQ. Taking this into account, simulations with the general pulmonary distribution model (Fig. 2) and a slow distribution rate, equal to a distribution half-life of 2 h, was performed. The sampling time points chosen, based on the plasma concentration time profile and the BAL LOQ, was reevaluated based on correspondence between the plasma concentrations in the early and the late sample and the extent of pulmonary concentrations < BAL LOQ at the time of sampling. Based on this a first sampling time at 1 h after dose was chosen. The late sample should be taken in the descending part of the time concentration profile, at a time point when the plasma concentration is corresponding (i.e. equal) to the plasma concentration at the first sampling time point and being > the BAL LOQ. In this case, 13 h was the corresponding time point to the 1 h early sample.

The characterization of pulmonary distribution of RIF and antibiotics aimed at the pulmonary tract in general has been heavily focused on the ratio between ELF, AC and plasma [1–4]. It is as however also interesting to describe the rate of distribution. We therefore assumed a fast and a slow distribution rate as two possible characteristics. In the fast scenario, the rate of distribution between plasma and the pulmonary tract, k, was 41.6 h^−1^ equivalent to an almost instantaneous distribution (1 min) of drug from plasma to the pulmonary tract. In the second scenario, a slow (2 h) distribution rate (k = 0.35 h^−1^) between plasma and the pulmonary tract was assumed. Inter individual variability (IIV) in the distribution extent parameter (R) was only estimated in the simulations of designs with 2 samples per subject, and then fixed to 30 %.

A number of different study scenarios (Online Resource 3) for the fast and slow pulmonary distribution characteristics were considered. The study scenarios were varied with regards to samples taken per subject (1 or 2) and sample size (10, 20, 30 or 50 subjects). For the 1 sample per subject design half of the total subjects were sampled at the early time point and the other half at the late time point. As the PK model by Clewe et al [9] included allometric scaling of clearance and plasma volume by fat free mass (FFM), each subject added to the datasets was given a weight and height based on mean and standard deviations from a standard, male, reference population [18]. Individual FFM values (*FFM*_*i*_), assuming a male study population, were calculated as:

4 $$FFM_{i} = \frac{{WHS_{MAX} \times HT^{2} \times WT}}{{WHS_{50} \times HT^{2} + WT}}$$

where the maximal weight height squared *(WHS*_*MAX*_*)* is 42.92 kg/m^2^ and *WHS*_*50*_ is 30.93 kg/m^2^.

The Stochastic Simulation and Estimation tool (SSE) as provided in the Perl speaks NONMEM (PsN) software, version 4.4.3 [19] together with the software NONMEM, version 7.3 (Icon Development Solutions, Ellicot City, USA) [20], was used to create 1000 datasets for each scenario, simulating individual plasma and pulmonary concentrations using the model (Fig. 2) using the first order conditional estimation method with interaction (FOCE INTER). Estimation of *k*, *R*, residual error, and where applicable the IIV in *R*, was the carried out using the simulated data. The plasma PK parameters including IIV estimates from the publication by Clewe et al [9] were fixed but the simulated plasma concentrations were retained in the model i.e. a PPP&D approach [21]. Relative bias (%) and rRMSE (%) in the parameter estimates were calculated according to Eqs. 5 and 6, respectively.

5 $$Relative \,bias = 100 \times \frac{1}{N}\mathop \sum \limits_{i} \frac{{est_{i} - true_{i} }}{{true_{i} }}$$

6 $$Relative\, root\, mean \,square \,error = 100 \times \surd \frac{1}{N}\mathop \sum \limits_{i} \frac{{(est_{i} - true_{i} )^{2} }}{{true_{i}^{2} }}$$

where est_i_ represents the estimated typical population parameter value and true_i_ represents the true typical population estimate for the parameter.

## Results

**Fig. 3:**
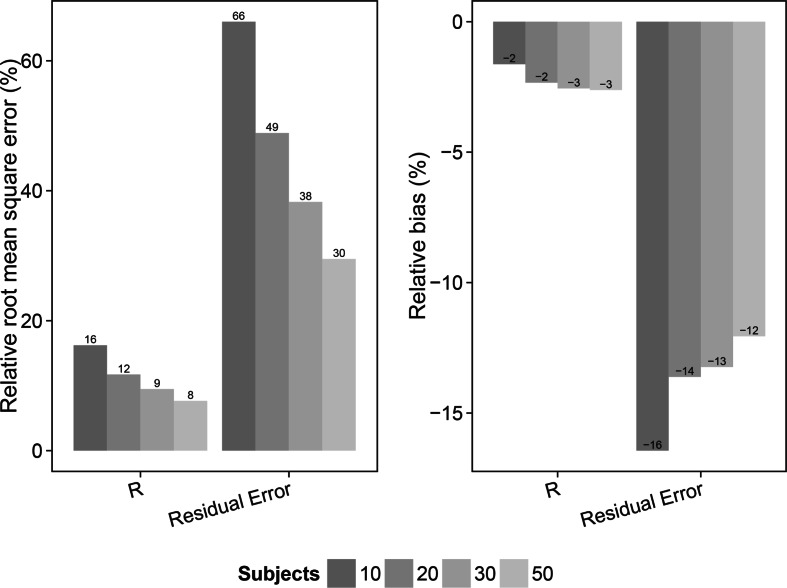
Relative root mean square error (rRMSE) (*left*) and relative bias (*right*) in the estimate of the bronchoalveolar lavage (BAL) fluid/plasma concentration distribution ratio (*R*) and the residual error for the scenarios with fast distribution and 1 sample per subject (scenarios 1–4). The different samples sizes are given in different *grey shades*

**Fig. 4:**
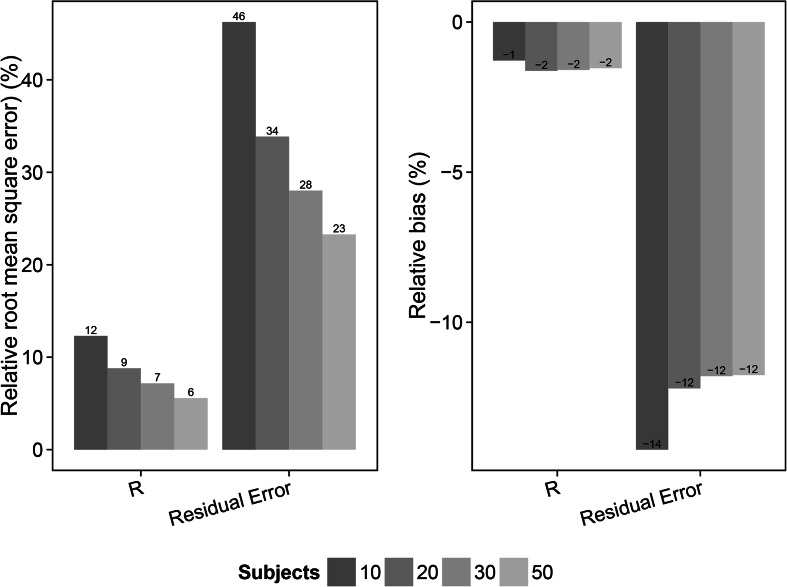
Relative root mean square error (rRMSE) (*left*) and relative bias (*right*) in the estimate of the bronchoalveolar lavage (BAL) fluid/plasma concentration distribution ratio (*R*) and the residual error for the scenarios with fast distribution and 2 sample per subject (scenarios 5–8). The different samples sizes are given in different *grey shades*

**Fig. 5:**
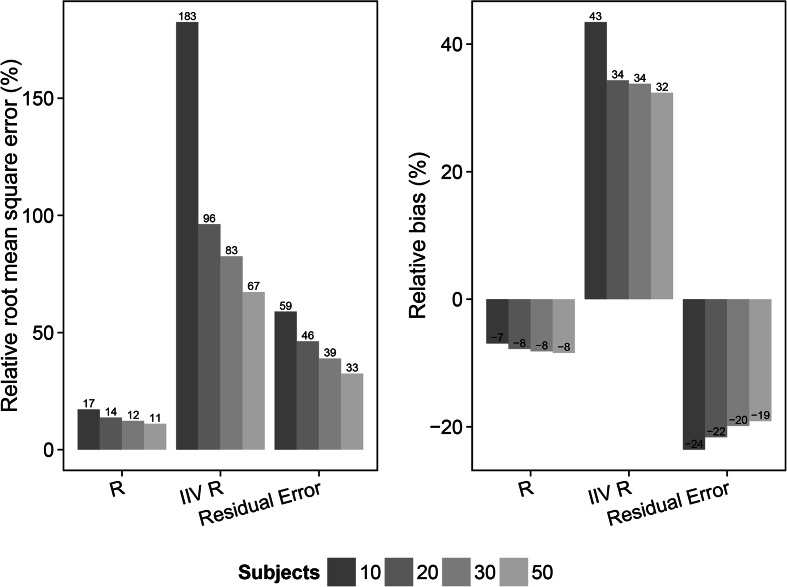
Relative root mean square error (rRMSE) (*left*) and relative bias (*right*) in the estimate of the bronchoalveolar lavage (BAL) fluid/plasma concentration distribution ratio (*R*), inter individual variability in the *R* parameter (IIV *R*) and the residual error for the scenarios with fast distribution and 2 sample per subject (scenarios 9–12). The different samples sizes are given in different *grey shades*

**Fig. 6:**
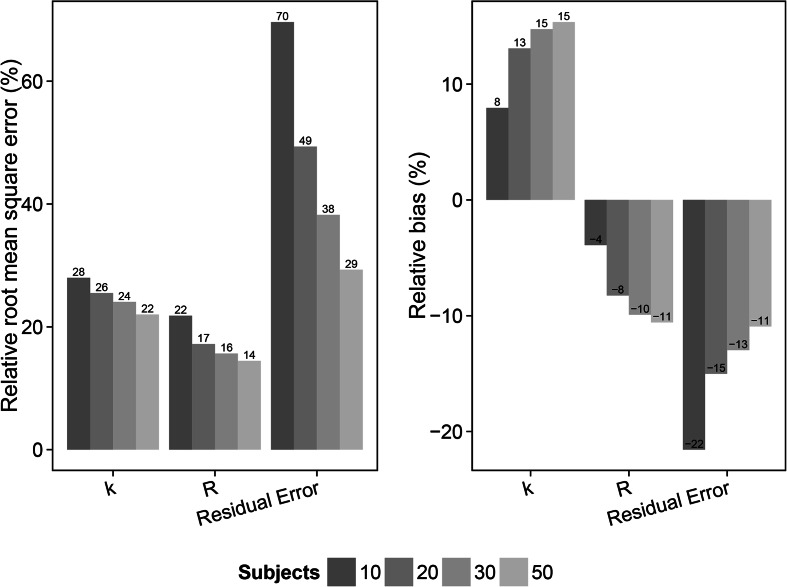
Relative root mean square error (rRMSE) (*left*) and relative bias (*right*) in the estimate of the distribution rate constant for the transfer of drug from the plasma to the bronchoalveolar lavage (BAL) fluid (*k*), the BAL fluid/plasma concentration distribution ratio (*R*) and the residual error for the scenarios with slow distribution and 1 sample per subject (scenarios 13–16). The different samples sizes are given in different *grey shades*

**Fig. 7:**
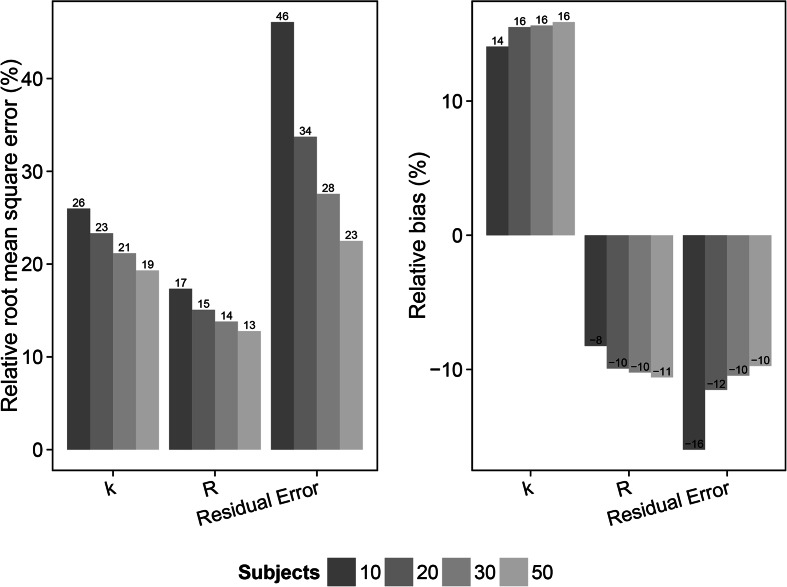
Relative root mean square error (rRMSE) (*left*) and relative bias (*right*) in the estimate of the distribution rate constant for the transfer of drug from the plasma to the bronchoalveolar lavage (BAL) fluid (*k*), the BAL fluid/plasma concentration distribution ratio (*R*) and the residual error for the scenarios with slow distribution and 2 sample per subject (scenarios 17–20). The different samples sizes are given in different *grey shades*

**Fig. 8:**
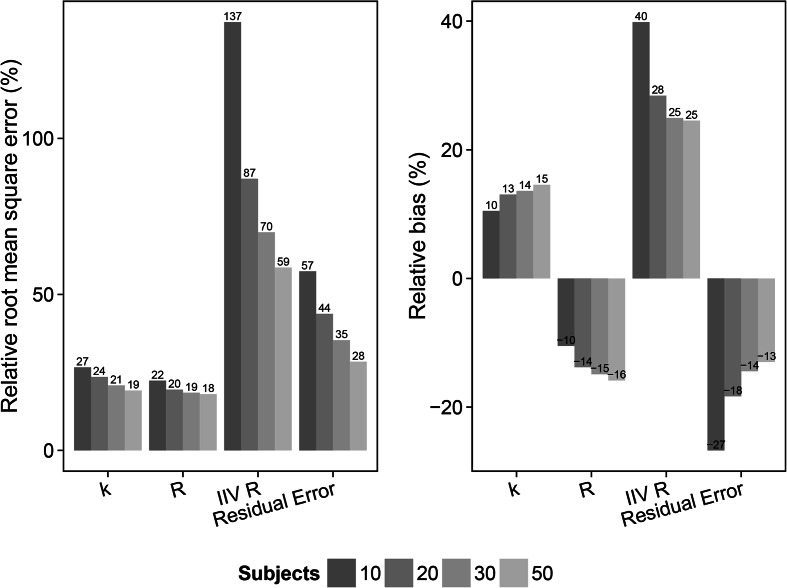
Relative root mean square error (rRMSE) (*left*) and relative bias (*right*) in the estimate of the distribution rate constant for the transfer of drug from the plasma to the bronchoalveolar lavage (BAL) fluid (*k*), the BAL fluid/plasma concentration distribution ratio (*R*), the inter individual variability in the *R* parameter (IIV *R*) and the residual error for the scenarios with slow distribution and 2 sample per subject (scenarios 21–24). The different samples sizes are given in different *grey shades*

The results from the evaluation, utilizing sampling time points at 1 and 13 h after dose, of the different scenarios are shown in Figs. 3, 4, 5, 6, 7, and 8 and in Online Resource 4. For the fast pulmonary distribution scenarios (1–12) using a 1 sample per subject design, both rRMSE and relative bias decreased for the parameter estimates of *R* and the residual error with increasing sample size. The rRMSE in *R* decreased from 16.2 % (10 subjects) to 7.7 % (50 subjects) and from 66 % (10 subjects) to 29.5 % (50 subjects) for the residual error. The relative bias in *R* increased slightly from −1.6 % (10 subjects) to −2.6 % (50 subjects) when increasing the number of subjects. The relative bias in the residual error decreased from −16.4 % (10 subjects) to −12.1 % (50 subjects). The evaluation using the same distribution rate (fast) but with 2 samples per subject shows similar trends but with an overall lower relative bias and rRMSE in the parameter estimates for the different study population sizes compared to the 1 sample per subject scenarios. For the 2 samples per subject scenarios (5–8), rRMSE decreases from 12.3 % (10 subjects) to 5.6 % (50 subjects) and from 46.2 % (10 subjects) to 23.3 % (50 subjects) in the estimation of *R* and the residual error, respectively. Relative bias in the *R* parameter increased slightly when increasing the sample size from 10 to 50 subjects, +0.02. The relative bias in the residual error decreased from from −14.2 % (10 subjects) to −11.8 % (50 subjects). Taking 2 samples per subjects with the fast distribution from plasma to ELF also allowed for including IIV on the *R* parameter (scenario 9–12). The rRMSE for the parameter estimates of *R*, IIV in *R* and the residual error decreased with increasing sample size; from 17.2 % (10 subjects) to 11.1 % (50 subjects), from 182.6 % (10 subjects) to 67.3 % (50 subjects) and from 58.9 % (10 subjects) to 32.5 % (50 subjects), respectively. Relative bias for the *R* parameter estimate for these scenarios showed a slight increase, from −6.9 % (10 subjects) to −8.5 % (50 subjects). The relative bias in the estimates of IIV in the *R* parameter decreased from 43.5 % (10 subjects) to 32.4 % (50 subjects) and the estimates of the residual error remained approximately −20 % for all sample sizes.

The result from the slow distribution scenarios (13–24) and the 1 sample per subject designs (scenarios 13–16) showed an overall decrease in rRMSE for the parameter estimates with increasing number of subjects. Relative bias for the *k* and *R* parameter estimates on the other hand increased following increased study population sizes. For these scenarios (13–24), estimation of the rate parameter *k* was possible in contrast to the fast distribution scenarios (1–12), and included in the evaluation. rRMSE decreased from 28 % (10 subjects) to 22 % (50 subjects), 21.8 % (10 subjects) to 14.5 % (50 subjects) and 69.6 % (10 subjects) to 29.3 % (50 subjects) for estimates of *k*, *R* and the residual error, respectively. Relative bias increased from 7.9 % (10 subjects) to 15.4 % (50 subjects) and −3.9 % (10 subjects) to −10.6 % (50 subjects) for estimates of *k* and *R*, respectively. Relative bias in the residual error decreased from −21.6 % (10 subjects) to −11 % (50 subjects). The slow distribution rate scenarios (13–24) were also evaluated using 2 samples per subject and varying the size of the study population (scenarios 17–20). The rRMSE decreased from 26 % (10 subjects) to 19.3 % (50 subjects), 17.3 % (10 subjects) to 13.8 % (50 subjects) and 46.1 % (10 subjects) to 22.5 % (50 subjects) for estimates of *k*, *R* and the residual error, respectively, and showing lower overall rRMSE values for the parameter estimates compared to the 1 sample per subject scenarios (13-16). Relative bias in the *k* and *R* parameter estimates increased from 14.1 % (10 subjects) to 15.9 % (50 subjects) and −8.3 % (10 subjects) to −10.6 (50 subjects). Relative bias for the residual error decreased with increased sample size from -16 (10 subjects) to −9.7 (50 subjects). The slow distribution with 2 samples per subjects was also evaluated with estimation of IIV in *R*. The rRMSE for the parameter estimates of *k*, *R*, IIV in R and the residual error decreased with increasing sample size; 26.6 % (10 subjects) to 19.2 % (50 subjects); 22.4 % (10 subjects) to 18 % (50 subjects); 137.3 % (10 subjects) to 58.6 % (50 subjects) and 57.4 % (10 subjects) to 28.5 % (50 subjects), respectively. The relative bias in the *k* and *R* parameter estimates increased from 10.5 % (10 subjects) to 14.6 % (50 subjects) and -10.5 % (10 subjects) to -15.9 % (50 subjects), respectively. Relative bias in the IIV of *R* parameter estimates decreased with increased number of subjects in the study population; 39.9% (10 subjects) to 24.5 % (50 subjects). A decrease in relative bias was also observed in the residual error, −26.7 % (10 subjects) to −13 % (50 subjects).

## Discussion

An optimized sampling design for BAL sampling have been developed and evaluated for two different distributions scenarios, fast and slow, with respect to the impact of different number of samples per subject and sample size. The sampling designs were investigated using a previously published pharmacometric modeling approach for describing pulmonary distribution [9]. The approach was carried out using a model (Fig. 2) that included a previously published RIF plasma PK model [17] and a general pulmonary distribution model (Eq. 3). Simulations with the model using different distribution rate constants (Online Resource 1) and distribution ratios (extents) (Online Resource 2) showed that although the model represents a simplistic approach to distribution characterization it is very much capable of handling different distribution properties such as different ELF and AC distribution rate constants and or distribution ratios (extents). In this study a general pulmonary distribution compartment (Eq. 3), referred to as BAL fluid, was used to represent a hypothetical scenario in which the distribution from plasma to ELF or AC is either similar or a scenario in which only ELF or AC is of interest. Evaluation of using joint or separate ELF and AC distribution rate constants and distribution ratios (extents) should naturally be explored during data analysis of observed clinical data.

The sampling design involves sampling at only 2 time points, one early and one late and can be performed with only 1 sample per subject in accordance with how most BAL studies are performed. The sampling approach relies on that the plasma concentration time profile of the studied drug as well as that the LOQ for the drug concentration in the BAL sample is known. In addition, a population pharmacokinetic model for plasma concentrations is needed. The time of sampling of the two samples is decided based on usage of the drug specific plasma PK model and needs to be exhibit correspondence in the early and the late sample with regards to plasma concentration. The time of the early sample should be as early as possible on the ascending part of the concentration time profile, to maximize the chance of capturing fast distribution rates from plasma to the pulmonary tract. However, one needs to consider that a slow distribution might require taking the first BAL sample at a later time point in order to reduce the risk of pulmonary concentrations < LOQ. This leads to that the late BAL sample will be taken earlier since it should be taken at a time point where the plasma concentrations for the BAL samples are the same. Further if the uncertainty in low concentrations is expected to be large it is naturally advisable to move the sampling time point further away from the LOQ. The late sample should be taken on the descending part of the concentration time profile at a time point where the plasma concentration is similar to that of the sample from the ascending part. In summary the decision of when to sample is carried in three steps:

1. A plasma concentration versus time profile is simulated with the drug specific plasma PK model
2. Pulmonary BAL concentrations is simulated using the general pulmonary model [9] and an assumed slow distribution rate (if the distribution is truly perfusion limited i.e. fast, this assumption will not impact the bias and precision in PK estimates as shown in this work)
3. One early and one late BAL sampling time points are identified using the simulated plasma and BAL concentrations versus time profiles and the BAL LOQ. The two BAL sampling time points should be taken when the plasma concentration is the same and when the BAL samples are >BAL LOQ

As with all sampling designs, ensuring that concentrations are above ≥LOQ at the time of sampling is important when utilizing the suggested sampling design. Perhaps even more so in this case as only two samples are taken for the whole study population. As described in the methods section, the early sample should be taken as early as possible, keeping in mind that the drug concentration still needs to be ≥LOQ. For drugs with a perfusion rate limiting distribution to the pulmonary tract, the raising part of the profile of ELF or AC concentrations will follow the plasma profile. For drugs with a permeability limited distribution to the pulmonary tract characterized with a slow distribution rate, this will however naturally require a later early sampling time to ensure pulmonary concentrations to be ≥LOQ. In Online Resource 1, the ELF or AC profiles for a pulmonary slow distribution with a *k* of 0.346 h is shown. This is equivalent to slow distribution where the distribution half-life is 2 h and steady-state in ELF would be reached after 8–10 h. With such a slow distribution, the maximal concentration in ELF is reached at approximately 8 h. Despite this slow distribution, the rate and extent of distribution can be described given the proposed design. Although less likely, in a scenario where the second sample at approximately 13 h is higher than at the first sample, the model approach [9] have no problems handling scenarios where ELF or AC concentrations are higher at the late sample time point compared to the early time point as the plasma profile is known and linked to the general pulmonary distribution model. The suggested sampling design together with the suggested modeling approach [9] is able to describe pharmacokinetic data after different types of administration and non-linear types of pulmonary distribution, for example concentration dependent distribution. In summary, the general pulmonary distribution model can estimate the extent and rate of distribution given two samples for any nonlinear pulmonary PK after any type of route of administration given that there is a well characterized plasma PK model for the route of administration. The second sample can be taken even though maximum pulmonary distribution has not yet occurred, as in the slow distribution scenario in this work, since it is coupled to a known plasma PK model. However, there are limitations with using the pulmonary distribution model. Multiphasic types of distribution can naturally not be described which require a multi-compartmental type of description, which is not supported by the approach or the sampling design. However, multi-compartmental models are very unlikely to be supported by the sparse data collected in BAL studies. Further, it is not very likely to believe that peripheral distribution within ELF or AC fluid to occur, which would justify multiple-compartmental models.

The evaluation showed that regardless of distribution rate the 2 samples per subject sampling design resulted in lower rRMSE for all the parameter estimates compared to the 1 sample per subject sampling design. Regardless of number of samples per subject, an increase of the sample size lead to a decrease in rRMSE in all parameter estimates. For the slow distribution scenarios (13–24), an increase in relative bias in the *k* and *R* parameter estimates was observed when increasing the sample size. This probably reflects a non-symmetric distribution of estimates around the point estimate. To explore the impact of variability, in any of the distribution characterizing parameters scenarios, on parameter precision scenarios including IIV was included in the sampling design evaluation. In the publication by Clewe et al [9], the data did not support any variability in either *R* or *k*. In order to explore the impact of variability in the extent of distribution (R) in this work, a coefficient of variation (CV) of 30 % was assumed in *R* for scenario (9–12, 21–24). The inclusion of IIV on *R* and not *k* was purely hypothetical and this should naturally be explored during data analysis of observed clinical data obtained after a BAL study. Further the scenarios that included estimation of IIV of the *R* parameter was only conducted for the 2 samples per subject design due to that at least 2 samples are needed to separate residual variability from IIV. As expected, the results from the evaluation with inclusion of IIV, for both the fast and slow distribution scenarios, showed similar trends with regards to decreasing rRMSE for the parameter estimates with increased sample size. For the slow distribution scenarios, an increase in bias for the *k* and *R* parameter estimates following increased sample size was observed. This information alone implies that larger sample sizes generate estimates further away from the true value. However, the relative bias has to be considered together with the precision of the estimates (rRMSE) in order to make a fair evaluation of the scenarios. In all scenarios where the relative bias in parameter estimates increased the rRMSE value decreased with increasing sample size.

The developed sampling design enables characterization of the rate and extent of distribution from plasma to the pulmonary tract making use of only one or two BAL samples per subject. The approach is further applicable to situations where little is known with regards to pulmonary distribution properties and relies only pre-characterized plasma PK and LOQ of the BAL technique. The approach was evaluated for a general plasma to pulmonary distribution but is applicable to distribution to either ELF, AC or both using adequate data, a proper plasma PK model and in accordance with the data analysis a proper set of pulmonary distribution models. The evaluation of the sampling design revealed that two samples per subject out-performs one sample per subject and that not surprisingly, an increase in the sample size decreases the relative RMSE in the parameter estimates. The results do however suggest that the presented sampling design provides adequate precision in the parameter estimates using only one sample per subject.

## Electronic supplementary material

Supplementary material 1 (DOCX 184 kb)

Supplementary material 2 (DOCX 174 kb)

Supplementary material 3 (DOCX 18 kb)

Supplementary material 4 (DOCX 28 kb)
