# Supplementary material for: Evaluation of optimized bronchoalveolar lavage sampling designs for characterization of pulmonary drug distribution
Source: J Pharmacokinet Pharmacodyn. 2015 Aug 28;42(6):699–708. doi: 10.1007/s10928-015-9438-9 (PMC4624821; doi:10.1007/s10928-015-9438-9)
Supplement: Supplementary file 1 — Supplementary material 1 (DOCX 184 kb) [file 10928_2015_9438_MOESM1_ESM.docx]

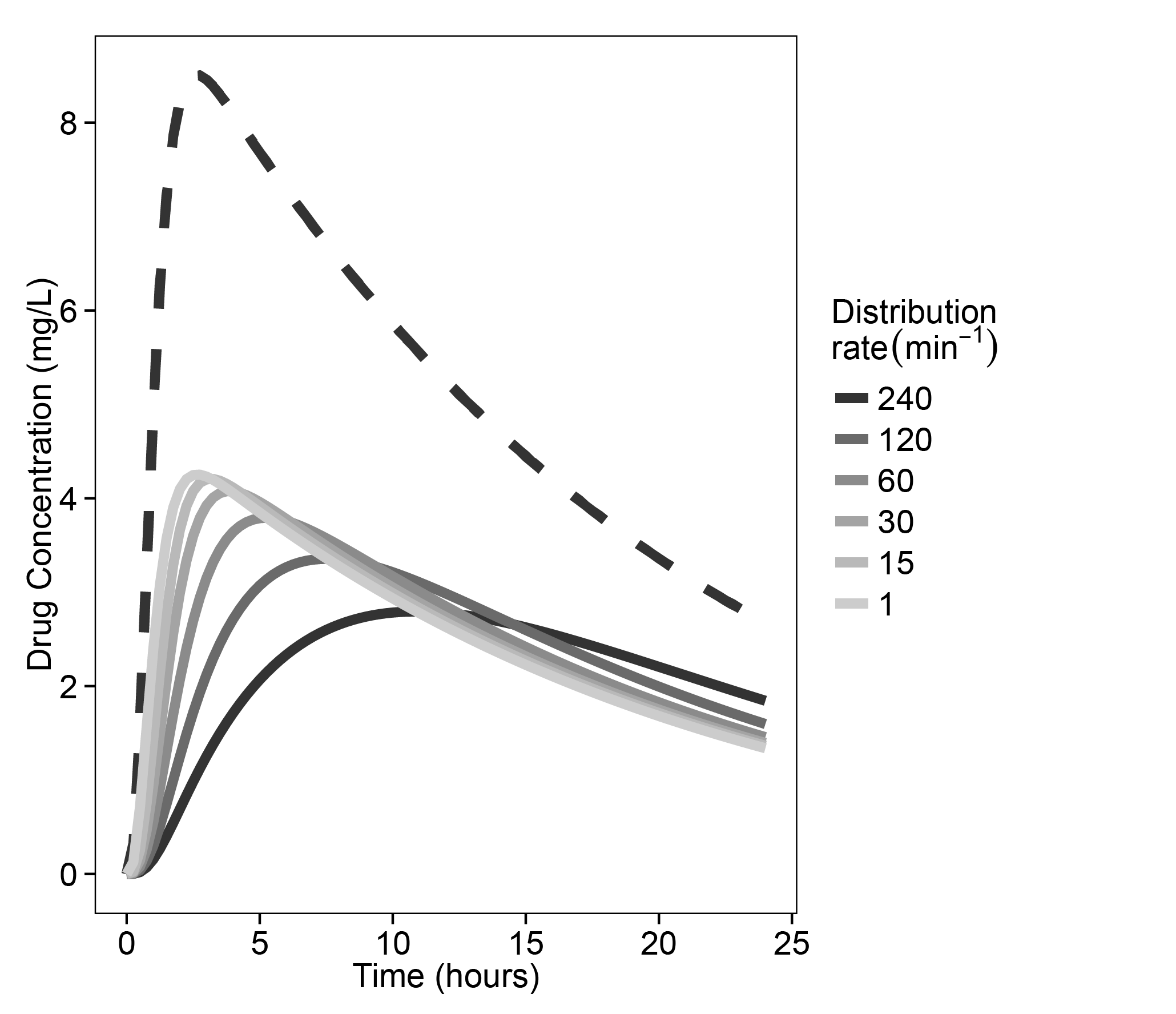


**Supplement Fig. 1**

Simulated typical drug concentration in bronchoalveolar lavage (BAL) fluid (solid lines) and plasma (dashed line) versus time after dose, using the general pulmonary distribution model [9] linked to a rifampicin plasma population pharmacokinetic model [17], for different pulmonary distribution rates (*k*) and a BAL fluid/plasma concentration distribution ratio (*R*) of 0.5. As can be seen in the figure, the general pulmonary distribution model, including only the parameters *R* and *k*, can describe a wide range of different pharmacokinetic profiles in epithelial lining fluid or alveolar cells.

Evaluation of Optimized Bronchoalveolar Lavage Sampling Designs For Characterization of Pulmonary Drug Distribution

Journal of Pharmacokinetics and Pharmacodynamics

Oskar Clewe^#^, Mats O. Karlsson and Ulrika S. H. Simonsson

Department of Pharmaceutical Biosciences, Uppsala University, Uppsala, Sweden

^#^Corresponding author

Mailing address: Department of Pharmaceutical Biosciences, BMC, Box 591, 751 24 Uppsala, Sweden

Email: oskar.clewe@farmbio.uu.se
