# Supplementary material for: Evaluation of optimized bronchoalveolar lavage sampling designs for characterization of pulmonary drug distribution
Source: J Pharmacokinet Pharmacodyn. 2015 Aug 28;42(6):699–708. doi: 10.1007/s10928-015-9438-9 (PMC4624821; doi:10.1007/s10928-015-9438-9)
Supplement: Supplementary file 3 — Supplementary material 3 (DOCX 18 kb) [file 10928_2015_9438_MOESM3_ESM.docx]

**ESM-3** Scenarios used in the design evaluation

| Scenario | k | IIV in R | Sample size | Samples/subject |
| --- | --- | --- | --- | --- |
| 1 | 1 minute | - | 10 | 1 |
| 2 | 1 minute | - | 20 | 1 |
| 3 | 1 minute | - | 30 | 1 |
| 4 | 1 minute | - | 50 | 1 |
| 5 | 1 minute | - | 10 | 2 |
| 6 | 1 minute | - | 20 | 2 |
| 7 | 1 minute | - | 30 | 2 |
| 8 | 1 minute | - | 50 | 2 |
| 9 | 1 minute | 30% | 10 | 2 |
| 10 | 1 minute | 30% | 20 | 2 |
| 11 | 1 minute | 30% | 30 | 2 |
| 12 | 1 minute | 30% | 50 | 2 |
| 13 | 2 hours | - | 10 | 1 |
| 14 | 2 hours | - | 20 | 1 |
| 15 | 2 hours | - | 30 | 1 |
| 16 | 2 hours | - | 50 | 1 |
| 17 | 2 hours | - | 10 | 2 |
| 18 | 2 hours | - | 20 | 2 |
| 19 | 2 hours | - | 30 | 2 |
| 20 | 2 hours | - | 50 | 2 |
| 21 | 2 hours | 30% | 10 | 2 |
| 22 | 2 hours | 30% | 20 | 2 |
| 23 | 2 hours | 30% | 30 | 2 |
| 24 | 2 hours | 30% | 50 | 2 |
|  |  |  |  |  |

IIV= inter-individual variability expressed as coefficient of variation, k= distribution rate constant for the transfer of drug from plasma to bronchoalveolar lavage (BAL) fluid, R = BAL/plasma concentration distribution ratio (extent)

Evaluation of Optimized Bronchoalveolar Lavage Sampling Designs For Characterization of Pulmonary Drug Distribution

Journal of Pharmacokinetics and Phramacodynamics

Oskar Clewe^#^, Mats O. Karlsson and Ulrika S. H. Simonsson

Department of Pharmaceutical Biosciences, Uppsala University, Uppsala, Sweden

^#^Corresponding author

Mailing address: Department of Pharmaceutical Biosciences, BMC, Box 591, 751 24 Uppsala, Sweden

Email: oskar.clewe@farmbio.uu.se
