# Supplementary material for: Evaluation of optimized bronchoalveolar lavage sampling designs for characterization of pulmonary drug distribution
Source: J Pharmacokinet Pharmacodyn. 2015 Aug 28;42(6):699–708. doi: 10.1007/s10928-015-9438-9 (PMC4624821; doi:10.1007/s10928-015-9438-9)
Supplement: Supplementary file 4 — Supplementary material 4 (DOCX 28 kb) [file 10928_2015_9438_MOESM4_ESM.docx]

**ESM-4** Simulation and estimation (SSE) results

| Scenario | Evaluation metric (%) | k | R | IIV R | Residual Error |
| --- | --- | --- | --- | --- | --- |
| 1 | Relative bias | - | -1.6 | - | -16.4 |
|  | rRMSE | - | 16.2 | - | 66 |
| 2 | Relative bias | - | -2.3 | - | -13.6 |
|  | rRMSE | - | 11.7 | - | 48.9 |
| 3 | Relative bias | - | -2.6 | - | -13.2 |
|  | rRMSE | - | 9.5 | - | 38.3 |
| 4 | Relative bias | - | -2.6 | - | -12.1 |
|  | rRMSE | - | 7.7 | - | 29.5 |
| 5 | Relative bias | - | -1.3 | - | -14.2 |
|  | rRMSE | - | 12.3 | - | 46.2 |
| 6 | Relative bias | - | -1.6 | - | -12.2 |
|  | rRMSE | - | 8.8 | - | 33.9 |
| 7 | Relative bias | - | -1.6 | - | -11.8 |
|  | rRMSE | - | 7.2 | - | 28 |
| 8 | Relative bias | - | -1.5 | - | -11.8 |
|  | rRMSE | - | 5.6 | - | 23.3 |
| 9 | Relative bias | - | -6.9 | 43.5 | -23.6 |
|  | rRMSE | - | 17.2 | 182.6 | 58.9 |
| 10 | Relative bias | - | -7.8 | 34.3 | -21.6 |
|  | rRMSE | - | 13.7 | 96.3 | 46.3 |
| 11 | Relative bias | - | -8.2 | 33.8 | -19.9 |
|  | rRMSE | - | 12.3 | 82.5 | 38.9 |
| 12 | Relative bias | - | -8.5 | 32.4 | -19.1 |
|  | rRMSE | - | 11.1 | 67.3 | 32.5 |
| 13 | Relative bias | 7.9 | -3.9 | - | -21.6 |
|  | rRMSE | 28 | 21.8 | - | 69.6 |
| 14 | Relative bias | 13.1 | -8.3 | - | -15 |
|  | rRMSE | 25.5 | 17.2 | - | 49.4 |
| 15 | Relative bias | 14.8 | -9.9 | - | -13 |
|  | rRMSE | 24.1 | 15.7 | - | 38.2 |
| 16 | Relative bias | 15.4 | -10.6 | - | -11 |
|  | rRMSE | 22 | 14.5 | - | 29.3 |
| 17 | Relative bias | 14.1 | -8.3 | - | -16 |
|  | rRMSE | 26 | 17.3 | - | 46.1 |
| 18 | Relative bias | 14.5 | -9.9 | - | -11.5 |
|  | rRMSE | 23.3 | 15.1 | - | 33.7 |
| 19 | Relative bias | 15.6 | -10.2 | - | -10.5 |
|  | rRMSE | 21.2 | 13.8 | - | 27.6 |
| 20 | Relative bias | 15.9 | -10.6 | - | -9.7 |
|  | rRMSE | 19.3 | 13.8 | - | 22.5 |
| 21 | Relative bias | 10.5 | -10.5 | 39.9 | -26.7 |
|  | rRMSE | 26.6 | 22.4 | 137.3 | 57.4 |
| 22 | Relative bias | 13.1 | -13.8 | 28.4 | -18.3 |
|  | rRMSE | 23.6 | 19.5 | 87.1 | 43.8 |
| 23 | Relative bias | 13.6 | -14.9 | 24.9 | -14.4 |
|  | rRMSE | 20.9 | 18.5 | 69.9 | 35.4 |
| 24 | Relative bias | 14.6 | -15.9 | 24.5 | -13 |
|  | rRMSE | 19.2 | 18 | 58.6 | 28.5 |

IIV= inter-individual variability expressed as coefficient of variation, k= distribution rate constant for the transfer of drug from plasma to bronchoalveolar lavage (BAL) fluid, R = BAL fluid/plasma concentration distribution ratio (extent), rRMSE= relative root mean square error

Evaluation of Optimized Bronchoalveolar Lavage Sampling Designs For Characterization of Pulmonary Drug Distribution

Journal of Pharmacokinetics and Pharmacodynamics

Oskar Clewe^#^, Mats O. Karlsson and Ulrika S. H. Simonsson

Department of Pharmaceutical Biosciences, Uppsala University, Uppsala, Sweden

^#^Corresponding author

Mailing address: Department of Pharmaceutical Biosciences, BMC, Box 591, 751 24 Uppsala, Sweden

Email: oskar.clewe@farmbio.uu.se
